# Susceptible window identification of the relationship between maternal ozone exposure and preterm birth

**DOI:** 10.1093/inthealth/ihaf073

**Published:** 2025-07-17

**Authors:** Kang-kang Zhong, Rui Yang, Xue-chun Liu, Jie He, Chuan-ting Wen, Zhi-wei Zhu, Ming-xuan Fan, Teng Bao, Qi Zhong

**Affiliations:** School of Public Health, Anhui Medical University, No. 81 Meishan Road, Shushan District, Hefei, Anhui Province, China; School of Public Health, Anhui Medical University, No. 81 Meishan Road, Shushan District, Hefei, Anhui Province, China; Department of Neurology, Second People's Hospital of Hefei, Hefei Hospital Affiliated to Anhui Medical University, No. 199 Changjiang East Road, Yaohai District, Hefei, Anhui Province, China; School of Public Health, Anhui Medical University, No. 81 Meishan Road, Shushan District, Hefei, Anhui Province, China; School of Public Health, Anhui Medical University, No. 81 Meishan Road, Shushan District, Hefei, Anhui Province, China; Jiangsu Province Hospital of Chinese Medicine, No. 155 Hanzhong Road, Gulou District, Nanjing, Jiangsu Province, China; School of Public Health, Anhui Medical University, No. 81 Meishan Road, Shushan District, Hefei, Anhui Province, China; Second School of Clinical Medicine, Anhui Medical University, 81 Meishan Road, Shushan District, Hefei, Anhui Province, China; School of Public Health, Anhui Medical University, No. 81 Meishan Road, Shushan District, Hefei, Anhui Province, China

**Keywords:** meta-analysis, O_3_ exposure, preterm birth, susceptible window

## Abstract

Previous studies have focused on the effects of ozone (O_3_) exposure and preterm birth (PTB), but the findings are contentious and the susceptible window for O_3_ exposure during pregnancy remains inconclusive. Here, we pooled the current evidence to explore the relationship between maternal O_3_ exposure and PTB and further identified the susceptible exposure windows. We pooled a meta-analysis of 17 eligible studies by searching Embase, PubMed and Web of Science through 9 September 2024. The odds ratio (OR) and the corresponding 95% confidence intervals (CIs) were extracted for analysis. The tests for heterogeneity, sensitivity and publication bias between studies were performed using Stata 15.0 (StataCorp, College Station, TX, USA). The combined results showed a positive association between O_3_ exposure and PTB (n=13; OR 1.065 [95% CI 1.056 to 1.073]), and middle pregnancy (gestational weeks 13–27) may be a susceptible window of O_3_ exposure with PTB (n=11; OR 1.033 [95% CI 1.029 to 1.036]). This meta-analysis suggested that O_3_ exposure during pregnancy may independently increase the risk of PTB and gestational weeks the 13–27 is a critical window for preventing this risk. Reducing outdoor activity or a wearing protective mask and multiple micronutrients supplementation during pregnancy may reduce the risk of O_3_ exposure in PTB.

## Introduction

Air pollution is one of the top five health risk factors, contributing to 6.67 million deaths worldwide in 2019.^1^ As a result, more scholars are paying attention to the harmful effects of air pollution on human health. In recent years, an increasing number of studies have focused on the adverse impacts of ambient air pollutants.^2^ These pollutants include particulate matter with aerodynamic diameters ≤2.5 μm (PM_2.5_) and ≤10 μm (PM_10_), as well as nitrogen dioxide (NO_2_), sulphur dioxide (SO_2_), carbon monoxide (CO) and ozone (O_3_).^2,3^

In recent years, O_3_ pollution has emerged as a critical concern for both the environment and public health. Economic growth and an increase in the number of motor vehicles have led to increasing O_3_ levels in many countries, especially in developing regions.^4^ As a powerful oxidizing agent, high concentrations of O_3_ can harm both vegetation and human health.^5^ Numerous epidemiological studies have shown that exposure to O_3_ is linked to various adverse health outcomes, including increased mortality rates, cardiovascular diseases, neurological disorders and respiratory diseases.^6,7^

Preterm birth (PTB) significantly increases the risk of neonatal mortality and is associated with a range of diseases during infancy and childhood.^8^ PTB places a greater economic burden on low- and middle-income countries compared with developed nations.^9^ Research shows that approximately 15 million preterm babies are born each year globally, resulting in a worldwide PTB rate of about 11%,^10^ with around 85% of these cases occurring in Asia and Africa.^11^ Therefore, controlling the incidence of PTB is an urgent priority in these regions.

The relationship between O_3_ exposure and PTB has been inconsistent in previous studies. A retrospective birth cohort study found that significant correlation between maternal O_3_ exposure and PTB (adjusted odds ratio [aOR] 1.023 [95% confidence interval {CI} 1.005 to 1.041]).^12^ However, another birth cohort study conducted in Tianjin, China, found a negative association between O_3_ exposure and PTB (hazard ratio [HR] 0.65 [95% CI 0.55 to 0.75]).^13^ Additionally, some studies from Canada, China, Italy and the USA have indicated no connection between maternal O_3_ exposure and PTB.^14–17^ The variability in findings may stem from the limitations of the covariates available in time-series ecological studies as well as the inability of cohort study designers to account for all possible confounding factors. This may result in differing conclusions across various studies. Therefore, it is important to note that some observed correlations may not imply causation. As a result, the causal relationship between maternal O_3_ exposure and PTB remains an unresolved issue.

Furthermore, if O_3_ exposure during pregnancy contributes to the risk of PTB, it is important to recognize that there are specific critical time windows during embryonic development when exposure may have varying effects.^18,19^ Previous studies have attempted to identify these sensitive exposure windows, but the results have often been inconsistent and limited. Generally, pregnancy is divided into three phases: the first, second and third trimesters.^20–22^ Most studies have employed relatively simple analytical methods, primarily examining the correlation between exposure levels and pregnancy outcomes without accounting for delayed effects.^23,24^ Recently, advancements in statistical methodologies, particularly distributed lag models, have improved the ability to pinpoint susceptible time windows for maternal exposure to air pollution and its associated adverse birth outcomes.^25,26^ Identifying the susceptible periods of maternal O_3_ exposure related to PTB is critically significant for clinical and public health applications. This knowledge can help inform effective antenatal care and clinical interventions while also increasing awareness of the potential relationships and mechanisms involved.^27^

To evaluate the susceptible period regarding the relationship between maternal O_3_ exposure and PTB, we conducted a systematic search for relevant studies and performed a quantitative analysis using a meta-analysis model. We expect that there will be growing public concern over the potential development of disorders in newborns due to O_3_ exposure during critical stages of pregnancy.

## Methods

### Search strategy

To thoroughly search for studies related to O_3_ exposure and PTB during pregnancy, we conducted a comprehensive review of the Embase, PubMed and Web of Science databases before 9 September 2024. The search terms we used included ‘ozone’, ‘stratospheric ozone’, ‘air pollution’ or ‘tobacco pollution’ AND ‘adverse pregnancy outcomes’, ‘pre-term birth’, ‘birth outcomes’ or ‘newborn outcomes’. Additionally, we manually screened all citations in the included articles and related reviews. Studies that met specific inclusion and exclusion criteria were taken into consideration.

To ensure that no references were overlooked, all related materials were manually reviewed by two authors (K-kZ and RY). Additionally, a double-check system was employed for the subsequent collation and analysis of data. In the event of any disagreements, a third investigator (JH) was consulted to help reach a final decision.

### Inclusion and exclusion criteria

Studies that met the following standards were eligible to be included in the meta-analysis: original complete article, O_3_ exposure during pregnancy, indicator effect sizes (ESs) and 95% CIs for inclusion effects, outcome indicators for assessing PTB and population studies. Exclusion criteria were not original literature or case reports, O_3_ exposure and PTB not reported, no ES value and 95% CI, no maternal exposure, animal studies and records of study subjects not conforming.

### Data acquisition and quality evaluation

After a full reading of the retained articles, data were extracted from those articles that met the criteria to make a feature table (Table 1). The following information was taken from each included article: first author of the research, country, study design, data year, sample size, maternal age, gestational period of exposure and O_3_ exposure concentration (μg/m^3^). If O_3_ concentrations were measured in parts per billion (ppb), we converted them to micrograms per cubic meter (μg/m³) (1 ppb=2.14 μg/m^3^). Because studies employing different exposure representations estimated distinct target parameters, making them uncomparable, we approached the data as follows: investigations were excluded from the study when estimating the target parameter in the entire pregnancy if that parameter was only assessed in one part of entire pregnancy (e.g.: early, mid or late pregnancy). Additionally, when the target parameter was evaluated in all three gestational periods but not across the entire pregnancy, we selected the highest parameter to include in the analysis of the entire pregnancy. Furthermore, when examining the association between PTB and O_3_ during each pregnancy, we included studies in our analyses only if they estimated the target parameter for at least one complete gestation. Articles containing inconsistent O_3_ exposure assessment methods in three trimesters were excluded. Generally, studies that included target parameters for the entire pregnancy or all three pregnancy periods were included in our study. We also included criteria for assessment of the outcome variable (PTB) and ESs and 95% CIs in the whole pregnancy or different periods of pregnancy.

**Table 1.** Baseline characteristics of the included studies.

Sources	Country (continent)	Sample size (n)	Data years	NOS score	Study design	Exposure period	Exposure (μg/m^3^)
Continuous							
Liu et al. 2003^31^	Canada (North America)	229 085	1985–1998	8	CS	Early and late	28.03
Hansen et al. 2006^32^	Australia (Oceania)	28 200	2000–2003	7	CS	Early and late	57.14
Lee et al. 2013^33^	USA (North America)	34 705	1997–2002	8	CS	Early	46.44
Olsson et al. 2012^21^	Sweden (Europe)	115 588	1988–1995	7	CS	Early and middle	55.30
Ha et al. 2014^34^	USA (North America)	423 719	2004–2005	8	CS	Whole	109.78
Zhengmin et al. 2016^24^	China (Asia)	95 911	2010–2013	7	CS	Whole	75.00
Mendola et al. 2016^35^	USA (North America)	223 502	2005–2007	8	CS	Whole	24.88
Lavigne et al. 2016^37^	Canada (North America)	818 400	2005–2012	6	CS	Whole	27.80
Gongbo et al. 2018^38^	Australia (Oceania)	24 702	2003–2013	8	CS	Whole	70.00
Juan et al. 2021^40^	China (Asia)	10 960	2014–2016	7	CS	Whole	98.20
Parra et al. 2021^41^	Brazil (South America)	979 306	2011–2016	7	CS	Whole	64.67
Jinwei et al. 2022^12^	China (Asia)	10 621	2018–2019	8	CS	Whole	92.13
Juan et al. 2023^13^	China (Asia)	70 760	2014–2018	8	CS	Whole	100.20
Qiong et al. 2018^54^	China (Asia)	469 975	2015–2017	8	CS	Whole	112.6
Categorical							
Capobussi et al. 2016^36^	Italy (Europe)	27 128	2005–2012	7	CS	Whole	58.20
Yingying et al. 2018^16^	China (Asia)	6693	2014–2016	6	CS	Whole	89.70
Siddika et al. 2019^39^	Finland (Europe)	2528	1984–1990	7	CS	Whole	57.54
Shuoxin et al. 2022^42^	China (Asia)	6640	2018–2019	8	CS	Whole	121.00

Continuous: O_3_ exposure as a continuous variable; categorical: O_3_ exposure as a categorical variable.

Informed consent was obtained for all the literature included in this work. The review relied on published data with prior ethical clearance. Our data adhere to the Preferred Reporting Items for Systematic Reviews and Meta-Analyses guidelines for systematic reviews. The data we used were completely anonymized and did not contain any critical information. Patient-level and maternal O_3_ exposure data were obtained from published articles. Pregnancy was divided into three periods: early, middle and late. Early pregnancy was defined as the first 12 weeks of pregnancy, middle was defined as weeks 13–27 and late was defined as ≥28 gestational weeks. PTB was defined as delivery before 37 completed weeks of gestation.^28,29^ Moreover, we evaluated the reliability and quality of all included articles by the Newcastle–Ottawa scale (NOS) score. The selected studies were classified as high (score ≥7), medium (score 5–6) or low quality (score <5).

### Statistical analysis

All meta-analyses were completed with Stata 15.0 (StataCorp, College Station, TX, USA). Relevant data were extracted from the included articles to create a database, which was analysed using the meta-analysis module of the Stata plugin.

We used the OR value to represent the joint association between maternal O_3_ exposure and PTB. The specific statistical methods employed were as follows: (1) O_3_ exposure levels were assessed using the median. (2) Heterogeneity test (Q-test): a fixed-effects model was utilized to merge studies when there was no heterogeneity among the included articles (I^2^<50% and p>0.1). Conversely, a random-effects model was chosen when heterogeneity was present.^30^ (3) Subgroup analysis: the literature was analysed based on the characteristics of the original studies. We stratified the data by potential confounders and calculated effect sizes and 95% CIs for each subgroup separately. (4) Sensitivity analysis: this analysis was conducted to identify potentially unstable factors in the meta-analyses and to evaluate the effect of publication bias on the overall results. (5) Test and correction for publication bias: we assessed publication bias through both qualitative and quantitative methods using Begg's funnel plot and Egger's linear regression. Data from the literature were evaluated for publication bias, with effect values recombined as necessary. We also explored the causes of significant publication bias in the results. Egger's linear regression was specifically used to determine whether there was asymmetry in the study results related to the magnitude of the effects observed.

## Results

### Study selection and characteristics

**Figure 1.**
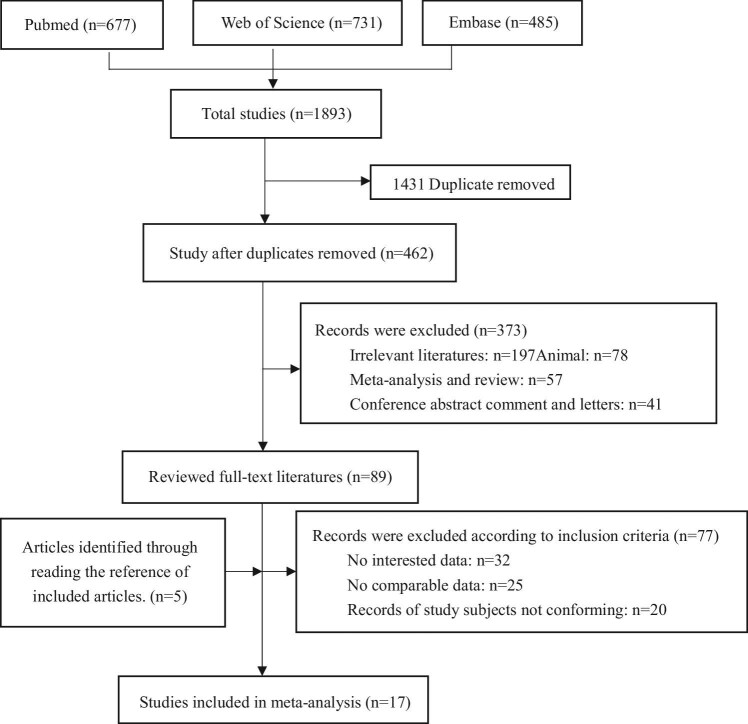
The flow chart of screening process.

A total of 462 unduplicated records were obtained using the search strategy in the three databases. After screening of titles and abstracts, 89 studies were chosen for complete-text reading. By reading the references of 89 documents, 5 were selected for inclusion. Finally, 17 studies assessing the association between O_3_ exposure and PTB were included in our meta-analysis (Figure 1).^12,13,21,24,31–42,54^

Selected studies were conducted in Asia (n=5), North America (n=4), Europe(n=1), Oceania (n=2) and South America (n=1). In the present study, 13 had an O_3_ exposure window of early pregnancy, 11 had an O_3_ exposure window of middle pregnancy and 12 had an O_3_ exposure window of late pregnancy and all had sufficient and complete data correlation to satisfy the follow-up analysis. According to the NOS score, 15 studies were ≥7 and were considered to be high-quality literature (Table 1).

### Association between maternal O_3_ exposure and PTB in different maternal periods

**Figure 2.**
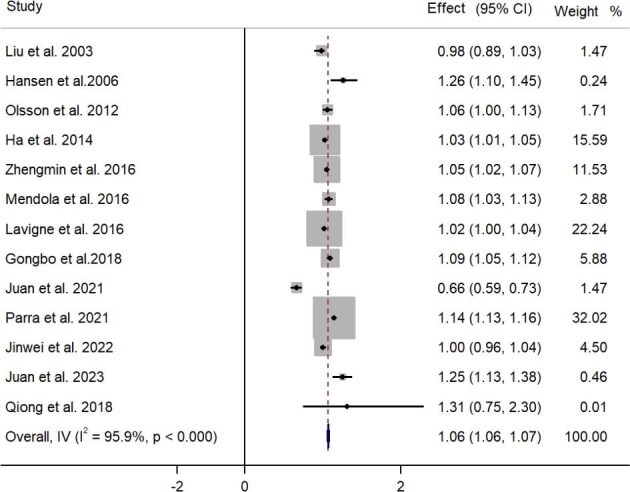
Forest plot of the association between maternal O_3_ exposure as a continuous variable with PTB in the whole pregnancy.

For this meta-analysis, we found a positive correlation between maternal O_3_ exposure as a continuous variable and PTB in the whole pregnancy (n=13; OR 1.061 [95% CI 1.053 to 1.069]; Figure 2). For O_3_ exposure in different maternal periods, the results showed negative correlations between maternal O_3_ exposure as a continuous variable in early pregnancy (n=13; OR 0.995 [95% CI 0.990 to 1.000]) and positive correlations in middle pregnancy (n=11; OR 1.033 [95% CI 1.029 to 1.036]; Table 2) and late pregnancy (n=12; OR 1.018 [95% CI 1.012 to 1.025]) with PTB (Supplementary Figure S1). We also found that the results of O_3_ exposure as a categorical variable with PTB were meaningless (Supplementary Figure S2). This indicates that middle pregnancy may be a susceptible window of the relationship between maternal O_3_ exposure and PTB. Moreover, infants who were born in North America or exposed to concentrations of O_3_ ≤64.37 μg/m^3^ may have a higher risk of PTB, especially during the susceptible period (Supplementary Table S2). Interventions and policies should be implemented to protect the health of pregnant women and infants, especially during the susceptible period.

**Table 2.** Subgroup analysis of the association between O_3_ exposure and PTB.

			Heterogeneity test			Publication bias	
Group	N	ES (95% CI)	Q	I^2^ (%)	p-Value	t	p-Value
Continuous							
Whole pregnancy	13	1.061 (1.053 to 1.069)	292.20	97.3	<0.001	−0.72	0.486
Early pregnancy	13	0.995 (0.989 to 1.000)	552.11	97.8	<0.001	−0.42	0.561
Middle pregnancy	11	1.026 (1.023 to 1.029)	408.50	97.8	<0.001	1.37	0.699
Late pregnancy	12	1.018 (1.012 to 1.029)	86.67	88.5	<0.001	1.92	0.409
Categorical							
Whole pregnancy	4	1.125 (0.926 to 1.324)	14.26	79.0	0.003	1.93	0.193
Early pregnancy	4	0.971 (0.764 to 1.117)	25.99	88.5	<0.001	0.16	0.928
Middle pregnancy	4	1.051 (0.854 to 1.248)	15.6	80.8	0.001	−1.77	0.171
Late pregnancy	4	0.937 (0.732 to 1.141)	19.06	84.3	<0.001	−0.23	0.739

Continuous: O_3_ exposure as a continuous variable; categorical: O_3_ exposure as a categorical variable.

### Subgroup analysis

Due to the large heterogeneity between the more frequent associations, we used subgroup analysis to explore the sources of heterogeneity, including the type of O_3_ exposure (continuous variable or categorical variable), data year (≤2015 or >2015), region (Asia, North America, South America, Oceania or Europe), concentrations of O_3_ exposure (≤64.37 μg/m^3^ or >64.37 μg/m^3^ for continuous variables and ≤77.71 μg/m^3^ or >77.71 μg/m^3^ for categorical variables), sample size (≤105 700 or >105 700 for continuous variables and ≤6677 or >6677 for categorical variables) and the timing of sample collection for exposure measures (early, middle or late pregnancy).

After performing subgroup analyses, O_3_ exposure was positively associated with PTB among studies conducted on O_3_ exposure as a continuous variable (OR 1.065 [95% CI 1.056 to 1.073]), in data year ≤2015 (OR 1.067 [95% CI 1.058 to 1.075]) and in North America (OR 1.028 [95% CI 1.015 to 1.041]) (Supplementary Table S1).

Since several of the above subgroups have a strong association between maternal O_3_ exposure and PTB, we analysed these subgroups in different trimesters (Supplementary Table S2).

### Sensitivity analyses

**Figure 3.**
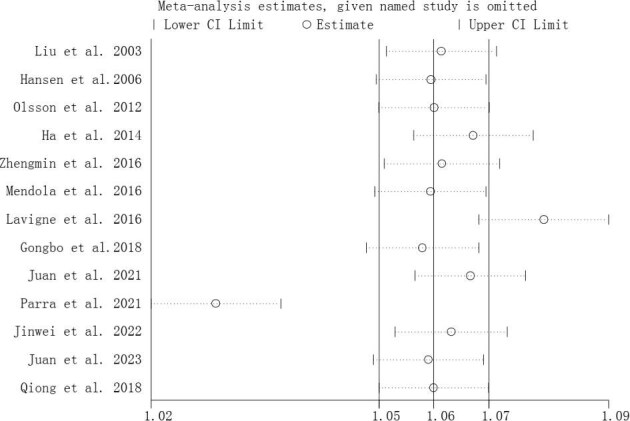
Sensitivity analysis of the association between O_3_ exposure as a continuous variable with PTB in the whole pregnancy.

A sensitivity analysis of the effects of maternal O_3_ exposure with PTB in different maternal periods by stepwise elimination showed that the correlation between maternal O_3_ exposure and PTB in the whole pregnancy (Figure 3) remained a positive association and the effect estimates remained relatively robust. The results of the sensitivity analyses in different periods of pregnancy are presented in Supplementary Figures S3 and S4).

### Publication bias

**Figure 4.**
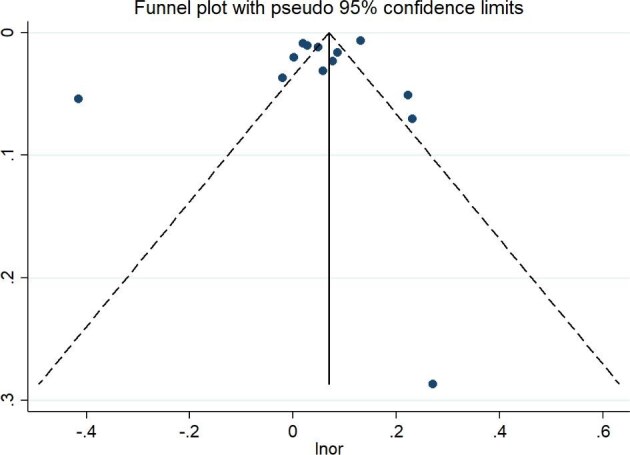
Funnel plot of the association between O_3_ exposure as a continuous variable with PTB in the whole pregnancy.

We used Begg's test and Egger's test to examine the publication bias of the literature (Figure 4, Supplementary Figures S5 and S6). We found no publication bias among all studies (p>0.05) (Supplementary Table S1).

## Discussion

This study evaluated the relationship between maternal O_3_ exposure and PTB during different stages of pregnancy. Our findings revealed a positive correlation between maternal O_3_ exposure and PTB. Middle pregnancy appears to be a particularly susceptible period for O_3_ exposure related to PTB. To reduce the risk of O_3_ exposure and PTB, it may be advisable for pregnant women to limit outdoor activities and wear protective mask.^53^

In recent years, O_3_ pollution has become a critical issue in both environmental and public health. Research strongly indicates that exposure to O_3_ can increase the risks of mortality and neurological diseases, particularly regarding adverse pregnancy outcomes.^43^ Pregnant women and foetuses are especially sensitive to hazardous environmental conditions, and several epidemiological studies have shown that O_3_ exposure is linked to various negative birth outcomes, including low birthweight (LBW), PTB and small for gestational age (SGA) infants.^44^ Currently, PTB is a widespread issue globally, with approximately 15 million preterm babies born each year, resulting in a global PTB rate of about 11%.^10^ Notably, around 85% of these cases occur in Asia and Africa.^11^ Thus controlling the incidence of PTB is an urgent priority in these regions. However, studies examining the relationship between O_3_ exposure and PTB have yielded conflicting results, and the sensitivity window for O_3_ exposure during pregnancy remains unclear. Our study found a positive correlation between maternal O_3_ exposure and PTB in middle pregnancy, which may be a particularly vulnerable period.

To explore potential sources of heterogeneity, we conducted subgroup analyses for each factor that might influence this meta-analysis. The results indicated that pollutant concentrations, gestational age at the time of sampling, maternal smoking status and geographic region were all potential factors affecting the relationship between maternal O_3_ exposure and PTB across different gestational periods. The correlations varied significantly by region, particularly in North America, where O_3_ exposure showed both positive and negative associations with changes in PTB rates. In our data, high O_3_ exposure levels (98.20 µg/m^3^ or 121.00 µg/m^3^) were recorded in China,^40,42^ where O_3_ exposure was found to be positively correlated with PTB rates. This discrepancy may be attributed to differences in industrial practices and O_3_ management across regions, leading to varying O_3_ concentrations to which pregnant women are exposed.

Additionally, the timing of sample collection plays a crucial role in the relationship between O_3_ exposure and PTB. The period from early to middle pregnancy is critical for organ development and functional initiation, establishing the foundations for foetal growth. This includes the onset of haematopoiesis, the formation of brown fat and secretion of thyroid hormones.^45^ This physiological phenomenon increases the exposure of both endogenous and exogenous substances in the foetus, including inflammatory substances and air pollutants.^46^ Recent studies indicate that exposure to environmental O_3_ during pregnancy is linked to an increased risk of PTB. However, the specific time frames during pregnancy when exposure is most harmful remain unclear. Identifying these critical windows of gestation could enhance our understanding of the potential mechanisms involved and help clarify the causal relationship between O_3_ exposure and PTB.

Many studies have indicated that exposure to ambient O_3_ during pregnancy is linked to an increased risk of PTB, although the specific windows of susceptibility vary.^47–49^ Early pregnancy, including the first and second trimesters, was generally found to be the susceptible exposure window,^44,50,51^ while some studies also reported associations for the third trimester, entire pregnancy or all three trimesters.^52,53^ In addition to gestation and throughout pregnancy, pre-pregnancy may also be a sensitive window of O_3_ exposure.^13^ Brauer et al.^14^ concluded that there is an inconsistent pattern regarding the most susceptible exposure window. They found a high correlation between exposures occurring during individual trimesters and those throughout the entire gestation period. This correlation complicates efforts to identify the specific time frames during pregnancy that are most vulnerable to particular pollutants. There are a few studies that have identified susceptible windows, which could provide evidence for future studies. The critical exposure window for high levels of O_3_ concentrations identified by Bai ^42^ after adjusting for an exposure matrix consisting of other pollutants and temperature was 13–18 weeks of pregnancy. The strongest correlation was also found at week 15 (O_3_=109.51 μg/m^3^; HR 1.06 [95% CI 1.01 to 1.13]). A retrospective cohort study conducted in Guangzhou, China, from 2015 to 2017 found that increased O_3_ concentrations in weeks 23–31 were associated with the risk of PTB (O_3_=112.60 μg/m^3^).^54^ The variation in the characteristics of the study populations, as well as the variations in O_3_ concentrations in different study areas, may cause inconsistencies in the critical window period. Thus we need to estimate the joint effect of mixtures and environmental factors, more closely reflecting the real-world impact of modulating between maternal O_3_ exposure and PTB.

We found that several factors, including temperature, socio-economic status (SES) and pregnancy complications, influence the incidence of PTB. A cohort study based on the National Birth Cohort Study in mainland China reported that both relatively low temperatures (below the 5th percentile, at 9.1°C) and high temperatures (above the 95th percentile, at 23.0°C) were associated with an increased risk of PTB when compared with the baseline temperature of 12°C.^55^ Additionally, Gray et al.^56^ reported that pregnant women with lower levels of education had a higher risk of the association between O_3_ and PTB (OR 1.05–1.12 for PTB). One study found that, when fully adjusted, there was an increased likelihood of PTB associated with a 10 μg/m³ increase in O_3_ and PM_10_ levels throughout pregnancy. Specifically, the ORs were 1.14 (95% CI 1.13 to 1.16) for O_3_ and 1.08 (95% CI 1.02 to 1.15) for PM_10_. The associations were influenced by maternal education levels and area-level SES for both pollutants. Mothers with lower educational attainment faced a higher risk of PTB, with an OR of 1.04 (95% CI 1.04 to 1.05). Similar modification effects were also observed for exposure to O_3_.^41^ Higher maternal education levels suggest a better awareness of the harmful effects of various environmental factors, healthier lifestyle choices and improved access to healthcare, which may mitigate the impact of air pollution on pregnancy outcomes.^57^ Women with lower levels of education are more likely to experience inadequate healthcare, nutritional deficiencies and unhealthy behaviours such as smoking^58^ that increase their susceptibility to air pollution and thus increase the risk of adverse pregnancy outcomes. Additionally, exposure to air pollution before pregnancy has been linked to higher levels of in utero inflammation and an increased risk of adverse birth outcomes, including PTB, LBW, SGA and large for gestational age.^40,59^ Previous studies have also shown that pregnant women with diabetes, asthma and pre-eclampsia have an increased risk of PTB. For PTB, O_3_-induced systemic inflammation and oxidative stress have been found to impair the function of the placenta and reduce transportation of oxygen and nutrients through the placenta.^60,61^ It also has been demonstrated that maternal O_3_ exposure induces systemic inflammation and oxidative stress as the primary potential mechanisms for adverse birth outcomes.^51,62^ However, the precise biological routes and the crucial molecular events during pregnancy with PTB remain unclear.

The following pathways may exist. On the one hand, short-^63^ and long-term^64^ O_3_ exposure leads to a significant increase in systemic inflammatory biomarkers (such as C-reactive protein and interleukin-6, among others), and the inflammatory reactions are risk factors for PTB.^65,66^ On the other hand, O_3_ exposure may lead to oxidative stress, which is associated with PTB.^67^ Women may be more sensitive to O_3_ exposure during ovulation and the 6- to 12-d post-fertilization phase of implantation. Moreover, unexpected pregnancies typically occur in young women.^68^ Women with unexpected pregnancies may not be fully aware of their condition and may not take necessary precautions at the beginning of the pregnancy. This lack of awareness makes them more susceptible to exposure to environmental risk factors. However, there have been few studies conducted on the effects of O_3_ exposure on germ cells and foetal development and the evidence supporting relevant mechanistic hypotheses is still insufficient. Therefore, further research is needed to validate the mechanisms by which O_3_ exposure is associated with PTB. For example, researchers could conduct experiments to uncover the causal relationship between O_3_ exposure and PTB by precisely timing the exposures during sensitive periods and then monitoring changes in biomarkers of the targeted areas.

We also found a number of interventions to reduce the risk of PTB. Bai et al.^42^ suggest that middle pregnancy is a sensitive window of exposure and intervention should be more important at this time. Strategies for pregnant women, like wearing protective masks outdoors, especially during the sensitive period, should be recommended to safeguard foetuses from the risks related to O_3_ exposure and PTB.^53^ A study found that pregnant women tend to limit outdoor activities on days with higher O_3_ concentrations, which can reduce the risk of PTB, particularly during sensitive periods (middle pregnancy).^69^ One review mentioned that multiple micronutrients supplementation significantly reduced the risk of PTB for women with a lower body mass index (BMI) but not among those with a higher BMI.^70^ In summary, interventions such as reducing outdoor activity or wearing protective equipment and MMN supplementation during pregnancy may reduce the risk of PTB to some extent. Therefore, our findings have considerable global public health implications for the development of management policies for O_3_ and interventions to prevent PTB.

The strengths of our study are as follows. First, this is the first meta-analysis assessing the correlation between maternal O_3_ exposure and PTB during different periods. Second, most of the included studies were retrospective cohort studies characterized by large sample sizes and high-quality, reliable results. Third, we concentrated on the sensitive period of O_3_ exposure in relation to PTB, during which specific interventions may effectively reduce the risk of PTB, and provide new insights into the underlying mechanisms.

Our study has several limitations. First, the small number of articles that specified the exact duration of O_3_ exposure may have led to biased results. Following our subgroup analysis, we found that the number of articles in the pregnant period subgroup was too small, making it difficult to adequately explain the source of heterogeneity. As a result, we focused on findings that were still supported by a sufficient amount of literature after stratifying the data. Second, we included fewer subgroups because most of the existing literature discussed various adjusting factors and the number of unadjusted articles was insufficient to support meaningful subgroup analyses. Consequently, the results did not provide strong evidence for specific indications. Lastly, our study only examined the effects of O_3_ exposure on PTB. However, adverse health effects are often linked to simultaneous exposure to multiple pollutants in real-life scenarios. Therefore, future research should place greater emphasis on joint analyses of multiple contaminants.

## Conclusions

In the present study, we found a correlation between O_3_ exposure and PTB during middle pregnancy, thus middle pregnancy may be a susceptible window. Study region, sampling time and O_3_ exposure concentrations were potential sources of heterogeneity among the included studies. Reducing outdoor activity or wearing protective equipment and MMN supplementation in pregnant women may reduce the risk of O_3_ exposure on PTB, especially during the susceptible period. Therefore, further interventions and policies are needed to reduce the harms of O_3_ exposure during pregnancy. Significantly, pregnant women are more sensitive to O_3_ exposure during middle pregnancy, which reminds that the relevant health authorities should strengthen the guidance and prenatal care to prevent the risk of PTB.

## Supplementary Material

ihaf073_Supplemental_File

## Data Availability

The data underlying this article will be shared upon reasonable request to the corresponding author.
