# Supplementary material for: Susceptible window identification of the relationship between maternal ozone exposure and preterm birth
Source: Int Health. 2025 Jul 17;18(2):208–17. doi: 10.1093/inthealth/ihaf073 (PMC13016642; doi:10.1093/inthealth/ihaf073)
Supplement: ihaf073_Supplemental_File [file ihaf073_supplemental_file.docx]

**Table S1 Subgroup analysis of the association between ozone exposure and preterm birth in whole pregnancy.**

| **Group** | **N** | **ES (95%CI)** | **Heterogeneity test** | | | **Publication bias** | |
| --- | --- | --- | --- | --- | --- | --- | --- |
|  |  |  | ***Q*** | ***I^2^(*%*)*** | ***p*** | ***t*** | ***p*** |
| **Continuous**  **Whole pregnancy** | 13 | 1.065(1.056, 1.073) | 292.48 | 95.9 | <0.001 | -0.72 | 0.486 |
| Data year(year) | 13 | 1.065(1.056, 1.073) | 292.48 | 95.9 | <0.001 | -0.72 | 0.486 |
| ≤2015 | 10 | 1.067(1.058, 1.075) | 274.02 | 96.7 | <0.001 | -0.99 | 0.350 |
| >2015 | 3 | 1.026(0.988, 1.064) | 14.24 | 86.0 | 0.001 | 0.93 | 0.522 |
| Area | 13 | 1.065(1.056, 1.073) | 292.48 | 95.9 | <0.001 | -0.72 | 0.486 |
| Asia  North America | 5  4 | 1.011(0.991, 1.031)  1.028(1.015, 1.041) | 120.75  7.52 | 96.7  60.1 | <0.001  0.057 | -0.43  4.70 | 0.696  0.042 |
| Oceania  South America  Europe | 2  1  1 | 1.097(1.062, 1.131)  1.140(1.130, 1.160)  1.060(1.000,1,130) | 3.49  /  / | 71.3  /  / | 0.062  /  / | /  /  / | /  /  / |
| Exposure (ug/m^3^) | 13 | 1.065(1.056, 1.073) | 292.48 | 95.9 | <0.001 | -0.72 | 0.486 |
| ≤64.37 | 5 | 1.088(1.077, 1.099) | 114.56 | 96.5 | <0.001 | -0.18 | 0.872 |
| >64.37 | 8 | 1.030(1.016, 1.043) | 135.39 | 95.6 | <0.001 | -0.41 | 0.696 |
| Sample size | 13 | 1.065(1.056, 1.073) | 292.48 | 95.9 | <0.001 | -0.72 | 0.486 |
| ≤105700  >105700 | 8  5 | 1.031(1.014, 1.048)  1.077(1.067, 1.086) | 146.00  126.86 | 95.2  96.1 | <0.001  <0.001 | -0.24  -0.18 | 0.816  0.864 |
| **Categorical**  **Whole pregnancy** | 4 | 1.125(0.926, 1.324) | 14.26 | 79.0 | 0.003 | 1.93 | 0.193 |
| Data year(year) | 4 | 1.125(0.926, 1.324) | 14.26 | 79.0 | 0.003 | 0.54 | 0.193 |
| ≤2015 | 2 | 1.004(0.781, 1.228) | 6.91 | 85.5 | 0.009 | / | / |
| >2015 | 2 | 1.579(1.146, 2.013) | 2.01 | 50.3 | 0.156 | / | / |
| Area  Asia  Europe | 4  2  2 | 1.125(0.926, 1.324)  1.579(1.146, 2.013)  1.004(0.781, 1.228) | 14.26  2.01  6.91 | 79.0  50.3  85.5 | 0.003  0.156  0.009 | 1.93  /  / | 0.193/  / |
| Exposure (ug/m^3^) | 4 | 1.125(0.926, 1.324) | 14.26 | 79.0 | 0.003 | 1.93 | 0.193 |
| ≤77.71 | 2 | 1.004(0.781, 1.228) | 6.91 | 85.5 | 0.009 | / | / |
| >77.71 | 2 | 1.579(1.146, 2.013) | 2.01 | 50.3 | 0.156 | / | / |
| Sample size | 4 | 1.125(0.926, 1.324) | 14.26 | 79.0 | 0.003 | 1.93 | 0.193 |
| ≤6677 | 2 | 2.083(1.475, 2.692) | 2.62 | 61.8 | 0.106 | / | / |
| >6677 | 2 | 1.011(0.800, 1.221) | 0.99 | 0.00 | 0.321 | / | / |

Abbreviations: Continuous, Ozone exposure as a continuous variable; Categorical, Ozone exposure as a categorical variable; ES, Effect size; “/”, No value.

**Table S2 Subgroup analysis of the association between ozone exposure and preterm birth in different period of pregnancy.**

| **Group** | **N** | **ES (95%CI)** | **Heterogeneity test** | | | **Publication bias** | |  |
| --- | --- | --- | --- | --- | --- | --- | --- | --- |
|  |  |  | ***Q*** | ***p*** | ***I^2^(*%*)*** | ***t*** | ***p*** |  |
| **Early pregnancy**  **Continuous**  Data year(year)  ≤2015  >2015  Area  Asia  North America  Oceania  Europe  South America  Exposure (ug/m^3^)  ≤64.37  >64.37  Sample size  ≤105700  >105700  **Early pregnancy**  **Categorical**  Data year(year)  ≤2015  >2015  Area  Asia  Europe  Exposure (ug/m^3^)  ≤77.71  >77.71  Sample size  ≤6677  >6677  **Middle pregnancy**  **Continuous**  Data year(year)  ≤2015  >2015  Area  Asia  North America  Oceania  Europe  South America  Exposure (ug/m^3^)  ≤64.37  >64.37  Sample size  ≤105700  >105700  **Middle pregnancy**  **Categorical**  Data year(year)  ≤2015  >2015  Area  Asia  Europe  Exposure (ug/m^3^)  ≤77.71  >77.71  Sample size  ≤6677  >6677  **Late pregnancy**  **Continuous**  Data year(year)  ≤2015  >2015  Area  Asia  North America  Oceania  South America  Exposure (ug/m^3^)  ≤64.37  >64.37  Sample size  ≤105700  >105700  **Late pregnancy**  **Categorical**  Data year(year)  ≤2015  >2015  Area  Asia  Europe  Exposure (ug/m^3^)  ≤77.71  >77.71  Sample size  ≤6677  >6677 | 13  13  10  3  13  5  4  2  1  1  13  6  7  13  7  6  4  4  2  2  4  2  2  4  2  2  4  2  2  11  11  8  3  11  5  3  1  1  1  11  4  7  11  6  5  4  4  2  2  4  2  2  4  2  2  4  2  2  12  12  9  3  12  5  4  2  1  12  5  7  12  7  5  4  4  2  2  4  2  2  4  2  2  4  2  2 | 0.995(0.990, 1.000)  0.995(0.990, 1.000)  0.991(0.984, 0.998)  0.999(0.992,1.006)  0.995(0.990, 1.000)  0.984(0.977, 0.990)  1.020(1.009, 1.031)  1.097(1.062, 1.131)  1.060(1.000, 1.130)  0.990(0.980, 1.000)  0.995(0.990, 1.000)  1.003(0.995, 1.011)  0.990(0.984, 0.996)  0.995(0.990, 1.000)  0.988(0.981, 0.994)  1.004(0.997, 1.012)  0.971(0.764, 1.117)  0.971(0.764, 1.117)  1.385(1.082, 1.689)  0.613(0.331, 0.895)  0.971(0.764, 1.117)  1.385(1.082, 1.689)  0.613(0.331, 0.895)  0.971(0.764, 1.117)  1.385(1.082, 1.689)  0.613(0.331, 0.895)  0.971(0.764, 1.117)  2.083(1.475, 2.692)  0.826(0.606, 1.046)  1.033(1.029, 1.036)  1.033(1.029, 1.036)  1.055(1.050, 1.059)  0.992(0.986, 0.998)  1.033(1.029, 1.036)  0.997(0.991, 1.003)  1.023(1.012, 1.034)  1.090(1.050, 1.120)  1.050(0.980, 1.120)  1.060(1.050, 1.060)  1.033(1.029, 1.036)  1.056(1.052, 1.061)  1.002(0.997, 1.008)  1.033(1.029, 1.036)  0.999(0.994, 1.005)  1.054(1.049, 1.058)  1.051(0.854, 1.248)  1.051(0.854, 1.248)  0.915(0.663, 1.168)  1.259(0.945, 1.573)  1.051(0.854, 1.248)  1.259(0.945, 1.573)  0.915(0.663, 1.168)  1.051(0.854, 1.248)  0.915(0.663, 1.168)  1.259(0.945, 1.573)  1.051(0.854, 1.248)  2.083(1.475, 2.692)  0.930(0.722, 1.138)  1.018(1.012, 1.025)  1.018(1.012, 1.025)  1.019(1.012, 1.025)  1.010(0.975, 1.046)  1.018(1.012, 1.025)  1.006(0.988, 1.025)  1.003(0.993, 1.014)  1.089(1.055, 1.123)  1.030(1.020, 1.040)  1.018(1.012, 1.025)  1.024(1.016, 1.033)  1.007(0.996, 1.018)  1.018(1.012, 1.025)  1.019(1.003, 1.035)  1.018(1.011, 1.026)  0.937(0.732, 1.141)  0.937(0.732, 1.141)  1.084(0.812, 1.335)  0.744(0.433, 1.055)  0.937(0.732, 1.141)  1.084(0.812, 1.335)  0.744(0.433, 1.055)  0.937(0.732, 1.141)  1.084(0.812, 1.335)  0.744(0.433, 1.055)  0.937(0.732, 1.141)  2.083(1.475, 2.692)  0.791(0.573, 1.008) | 547.28  547.28  514.04  30.81  547.28  471.09  4.43  3.49  /  /  547.28  29.99  511.16  547.28  513.18  23.05  25.99  25.99  3.46  9.20  25.99  3.46  9.20  25.99  3.46  9.20  25.99  2.62  8.87  318.88  318.88  47.87  0.42  318.88  43.37  0.31  /  /  /  318.88  20.71  77.74  318.88  69.87  37.07  15.60  15.60  7.42  5.39  15.60  5.39  7.42  15.60  7.42  5.39  15.60  8.87  0.64  87.43  87.43  84.76  2.46  87.43  46.72  9.61  0.10  /  87.43  9.84  71.67  87.43  68.76  18.66  19.06  19.06  8.00  8.46  19.06  8.00  8.46  19.06  8.00  8.46  19.06  2.62  1.07 | <0.001  <0.001  <0.001  <0.001  <0.001  <0.001  0.219  0.062  /  /  <0.001  <0.001  <0.001  <0.001  <0.001  <0.001  <0.001  <0.001  0.063  0.002  <0.001  0.063  0.002  <0.001  0.063  0.002  <0.001  0.106  0.003  <0.001  <0.001  <0.001  0.810  <0.001  <0.001  0.854  /  /  /  <0.001  <0.001  <0.001  <0.001  <0.001  <0.001  0.001  0.001  0.006  0.020  0.001  0.020  0.006  0.001  0.006  0.020  0.001  0.003  <0.001  <0.001  <0.001  <0.001  0.292  <0.001  <0.001  0.022  0.755  /  <0.001  0.043  <0.001  <0.001  <0.001  <0.001  <0.001  <0.001  0.005  0.004  <0.001  0.005  0.004  <0.001  0.005  0.004  <0.001  0.106  0.300 | 97.8  97.8  98.2  93.5  97.8  99.2  32.3  71.3  /  /  97.8  83.3  98.8  97.8  98.8  78.3  88.5  88.5  71.1  89.1  99.5  71.1  89.1  99.5  71.1  89.1  99.5  61.8  88.7  96.9  96.9  85.4  0.0  96.9  90.8  0.0  /  /  /  96.9  85.5  92.3  96.9  94.3  86.5  80.8  80.8  86.5  81.5  80.8  81.5  86.5  80.8  86.5  81.5  80.8  88.7  0.0  87.4  87.4  90.6  18.8  87.4  91.4  68.8  0.0  /  87.4  59.3  91.6  87.4  91.3  78.6  84.3  84.3  87.5  88.2  84.3  87.5  88.2  84.3  87.5  88.2  84.3  61.8  6.9 | 0.54  0.54  -0.18  1.34  0.54  -0.09  -0.41  /  /  /  0.54  1.82  0.12  0.54  0.19  2.02  0.10  0.10  /  /  0.10  /  /  0.10  /  /  0.10  /  /  0.39  0.39  -0.55  2.06  0.39  1.74  /  /  /  /  0.39  -1.22  2.75  0.39  3.00  -1.48  2.10  2.10  /  /  2.10  /  /  2.10  /  /  2.10  /  /  -0.48  -0.48  -0.69  2.86  -0.48  -0.32  -0.83  -0.83  /  -0.48  -1.46  0.28  -0.48  -0.95  -0.05  0.38  0.38  /  /  0.38  /  /  0.38  /  /  0.38  /  / | 0.602  0.602  0.866  0.408  0.561  0.935  0.719  /  /  /  0.602  0.143  0.909  0.602  0.855  0.113  0.928  0.928  /  /  0.928  /  /  0.928  /  /  0.928  /  /  0.706  0.706  0.601  0.288  0.706  0.179  /  /  /  /  0.706  0.347  0.040  0.706  0.058  0.213  0.171  0.171  /  /  0.171  /  /  0.171  /  /  0.171  /  /  0.640  0.640  0.513  0.214  0.640  0.771  0.492  /  /  0.640  0.242  0.789  0.640  0.387  0.961  0.739  0.739  /  /  0.739  /  /  0.739  /  /  0.739  /  / |  |

Abbreviations: Continuous, Ozone exposure as a continuous variable; Categorical, Ozone exposure as a categorical variable; ES, Effect size; “/”, No value.

**
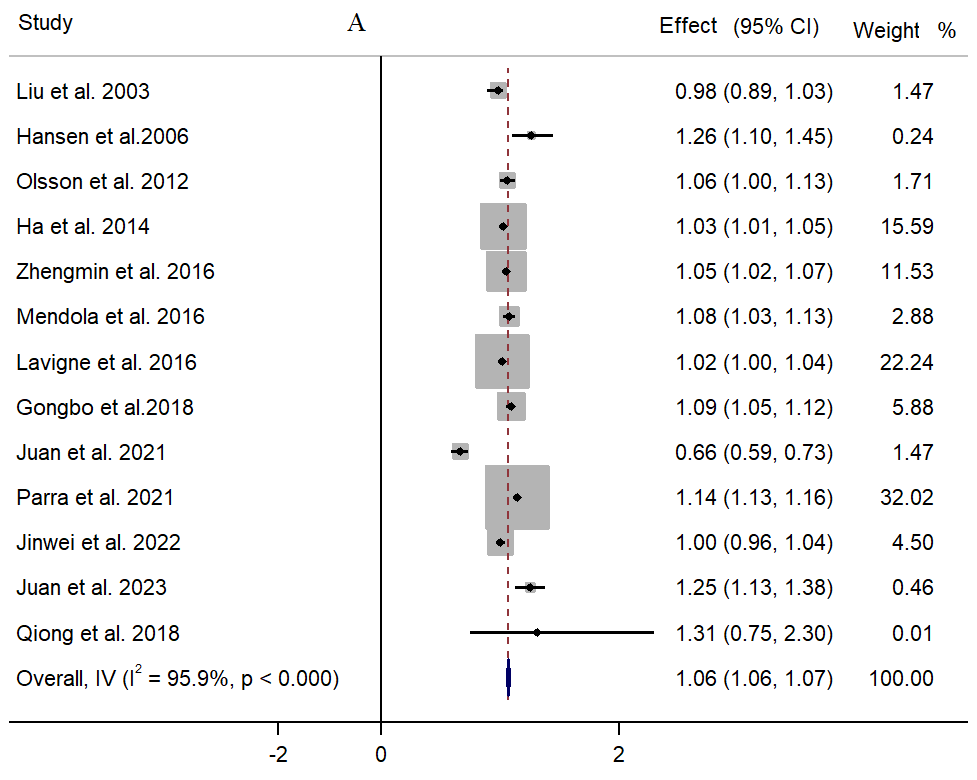

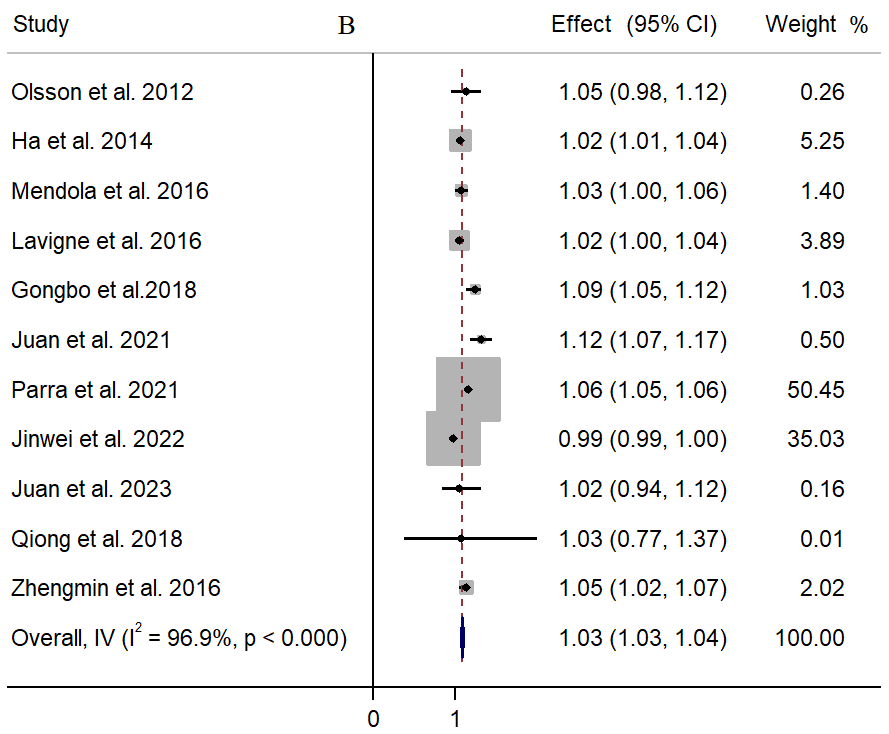
**

**
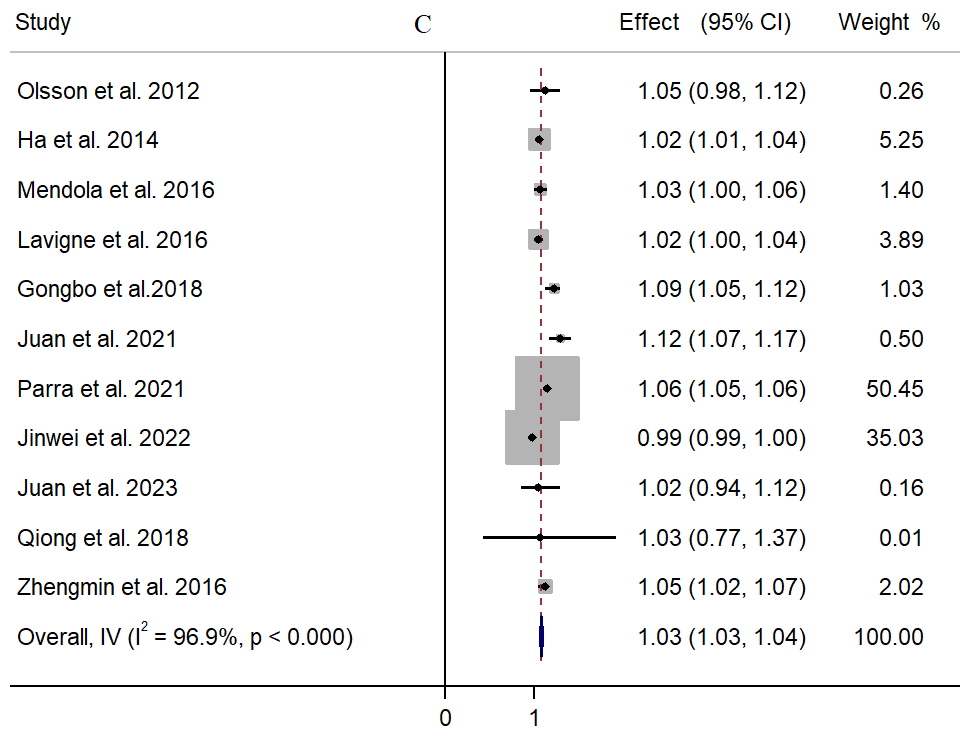

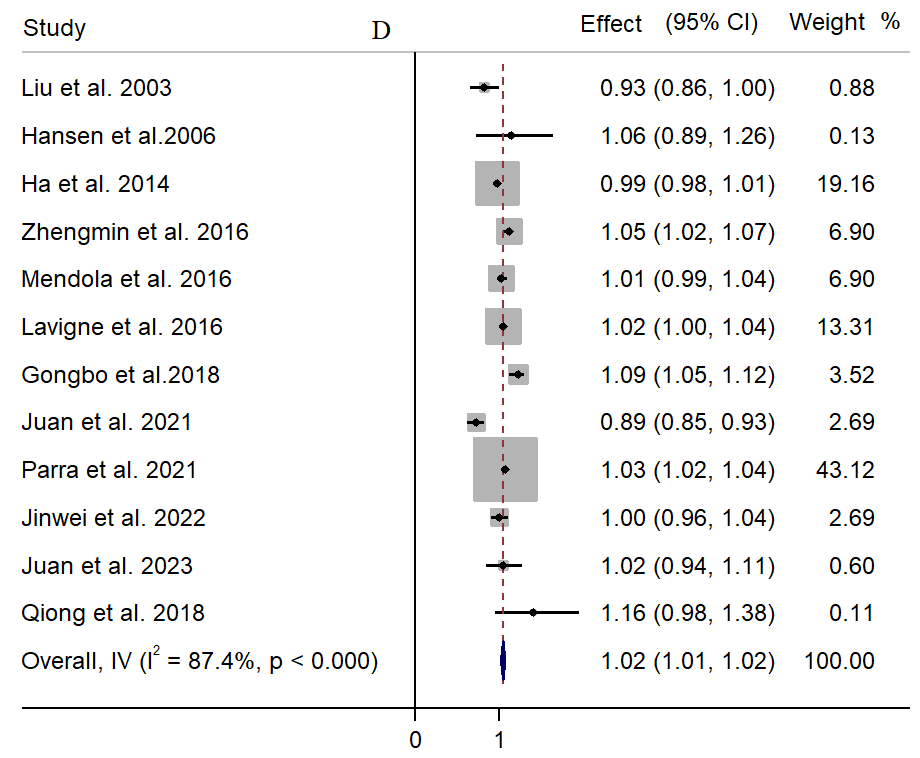
**

**Figure S1 Forest plot of the association between ozone exposure and preterm birth in different period of pregnancy. (A) Ozone exposure as a continuous variable in whole pregnancy; (B) Ozone exposure as a continuous variable in early pregnancy; (C) Ozone exposure as a continuous variable in middle pregnancy; (D) Ozone exposure as a continuous variable in late pregnancy.**

**
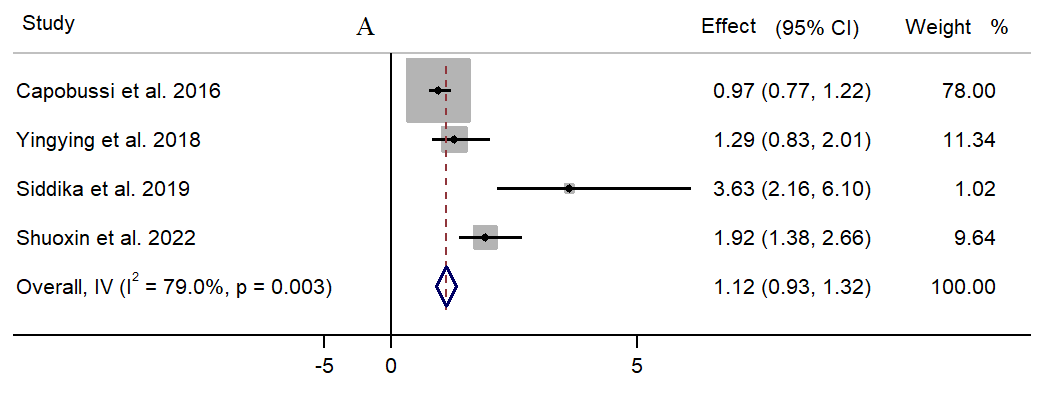

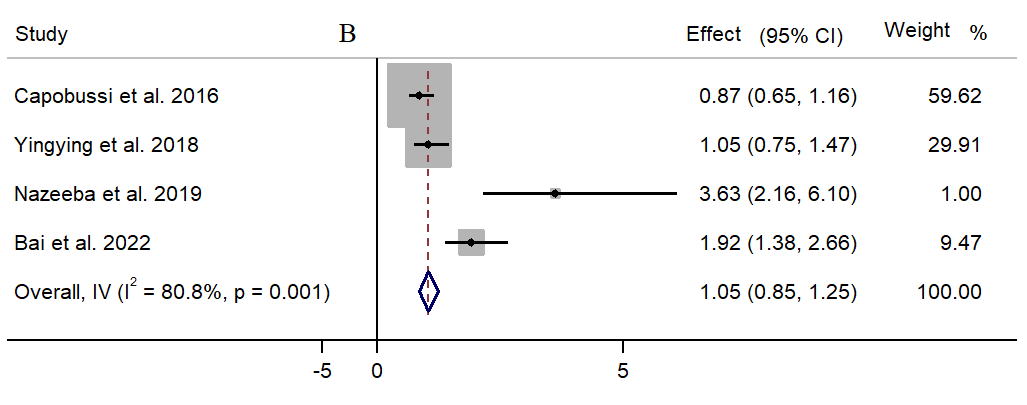
**

**
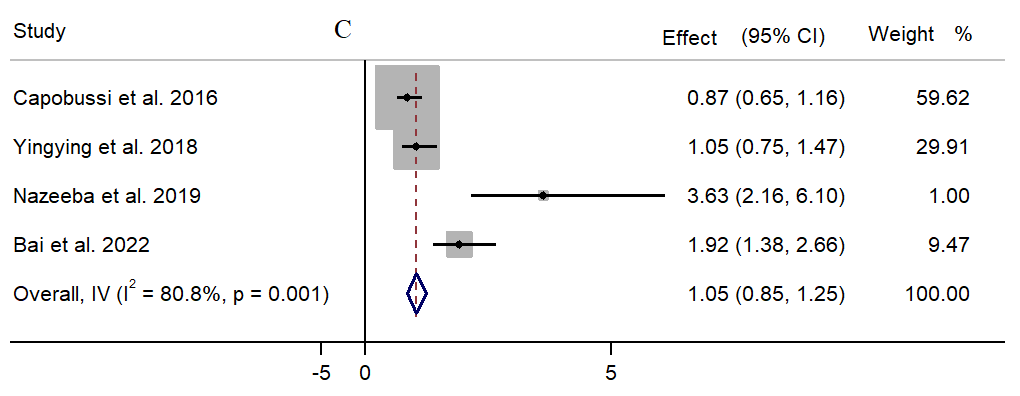

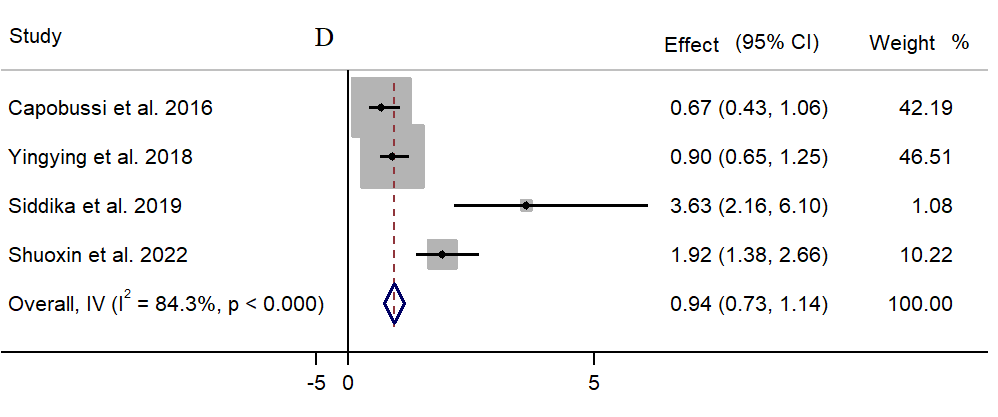
**

**Figure S2 Forest plot of the association between ozone exposure and preterm birth in different period of pregnancy. (A) Ozone exposure as a categorical variable in whole pregnancy; (B) Ozone exposure as a categorical variable in early pregnancy; (C) Ozone exposure as a categorical variable in middle pregnancy; (D) Ozone exposure as a categorical variable in late pregnancy.**


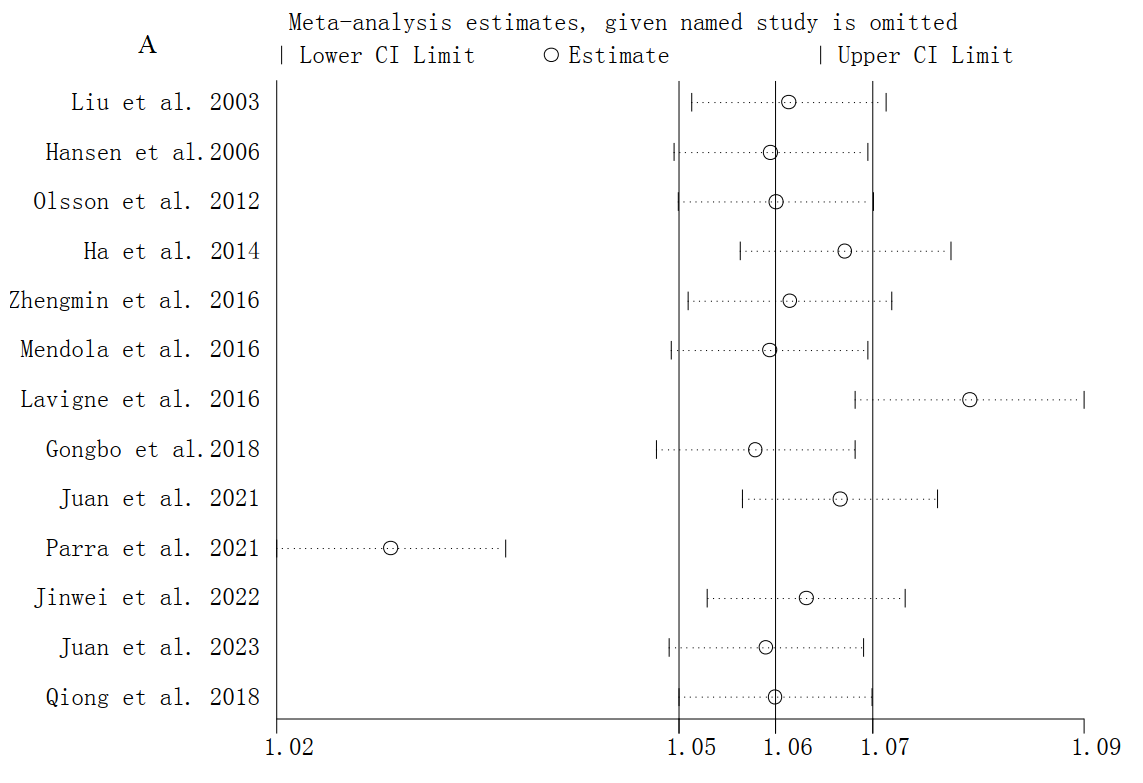

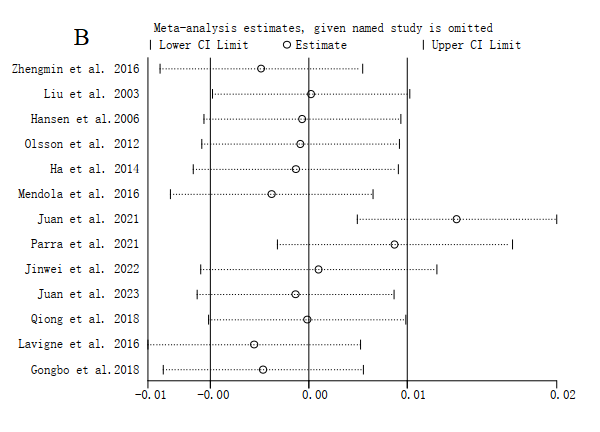


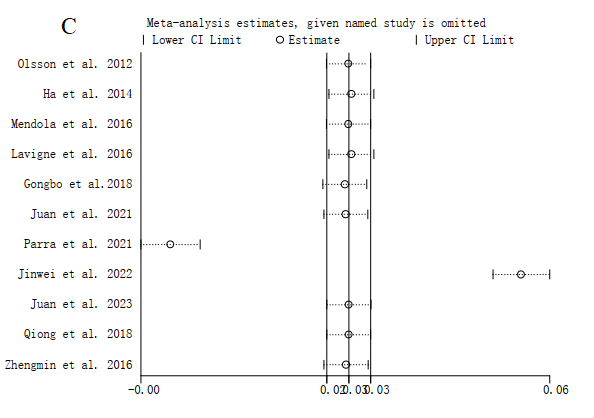

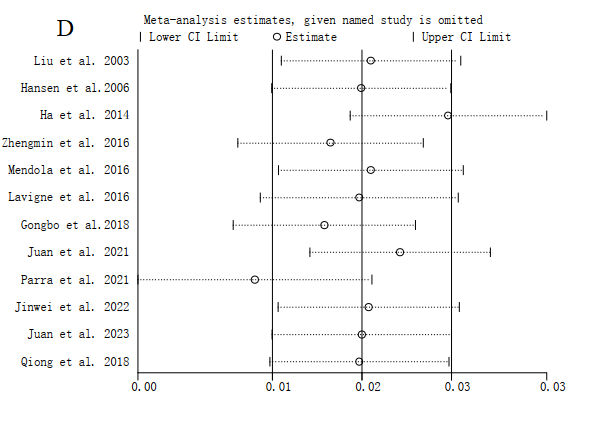


**Figure S3 Sensitivity analysis of the association between ozone exposure and preterm birth in pregnancy. (A) Ozone exposure as a continuous variable in whole pregnancy; (B)Ozone exposure as a continuous variable in early pregnancy; (C)Ozone exposure as a continuous variable in middle pregnancy; (D)Ozone exposure as a continuous variable in late pregnancy.**


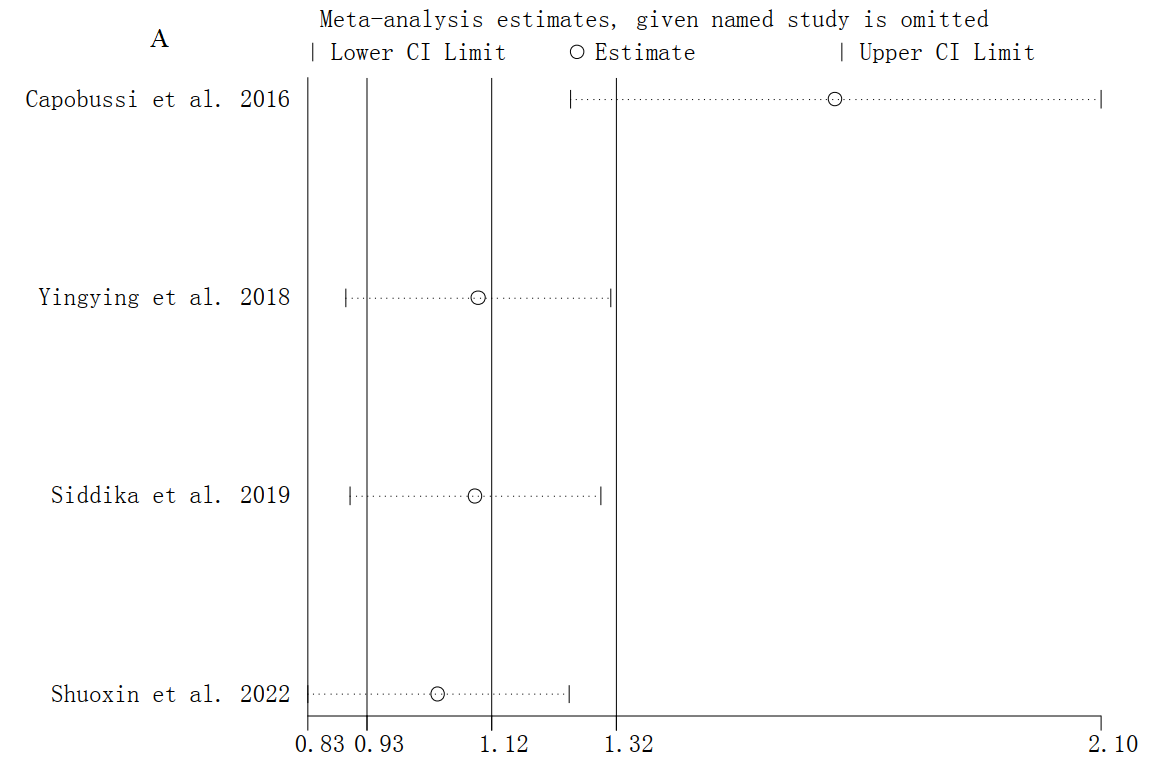

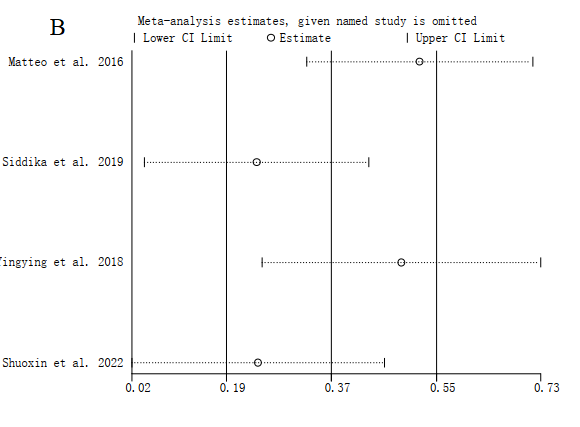


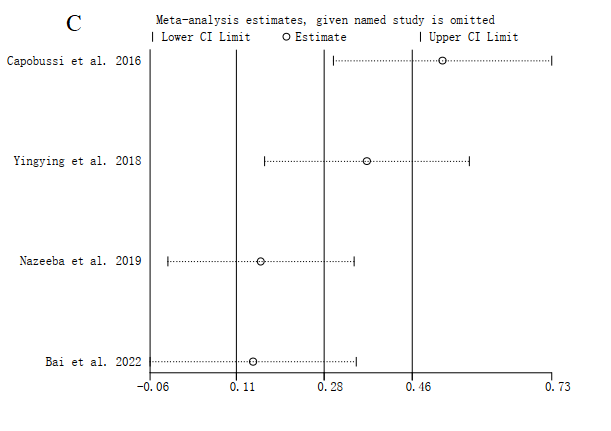

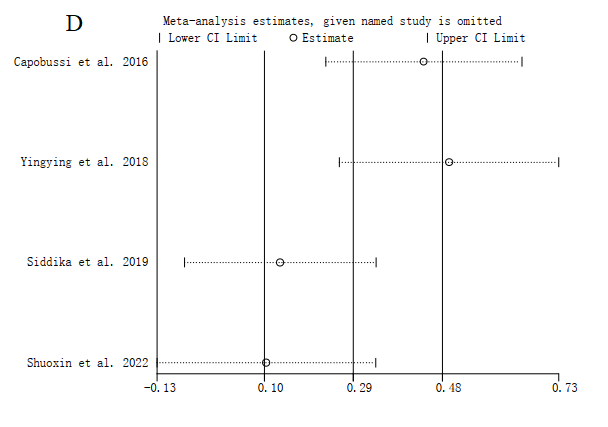


**Figure S4 Sensitivity analysis of the association between ozone exposure and preterm birth in pregnancy. (A) Ozone exposure as a categorical variable in whole pregnancy; (B) Ozone exposure as a categorical variable in early pregnancy; (C) Ozone exposure as a categorical variable in middle pregnancy; (D) Ozone exposure as a categorical variable in late pregnancy.**


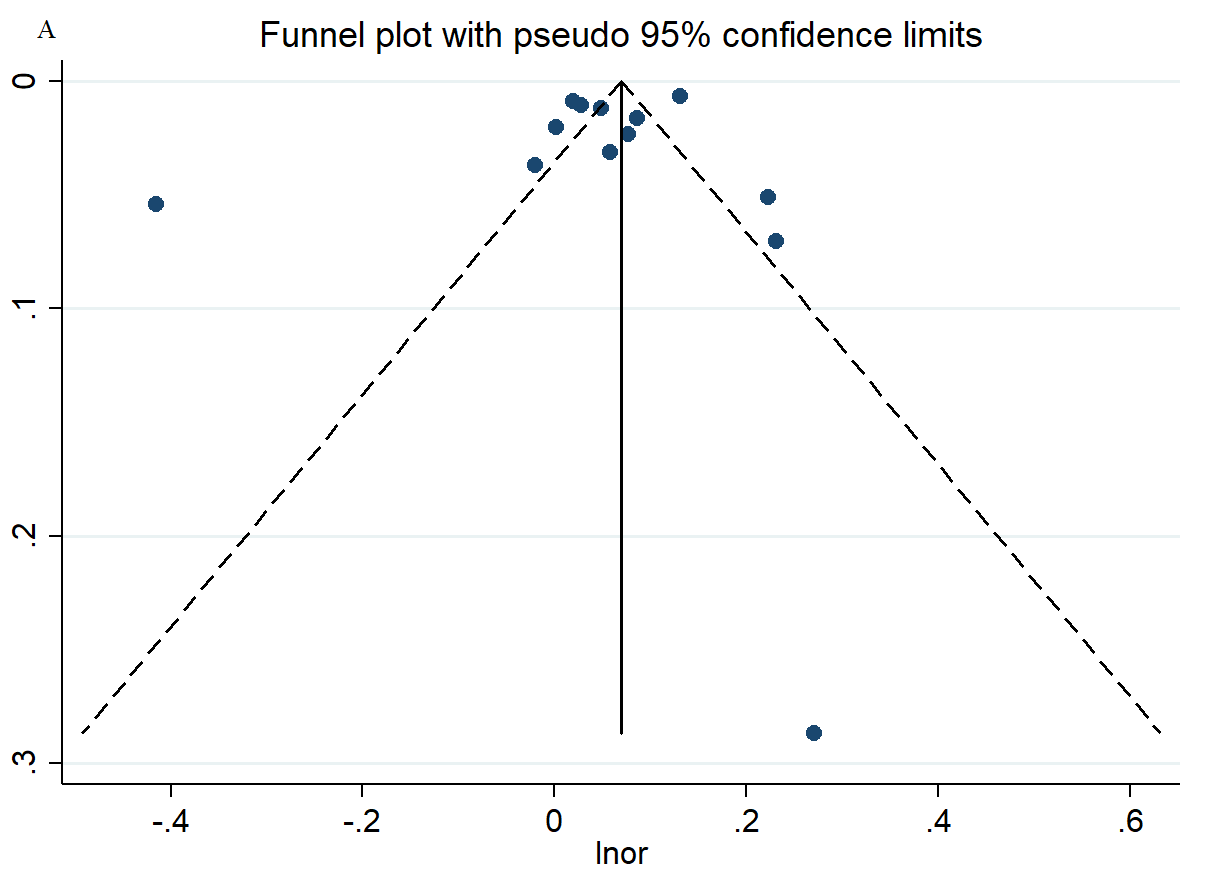

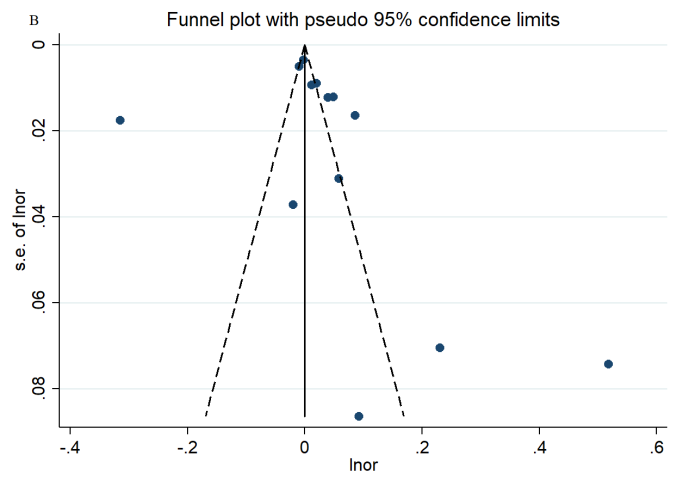


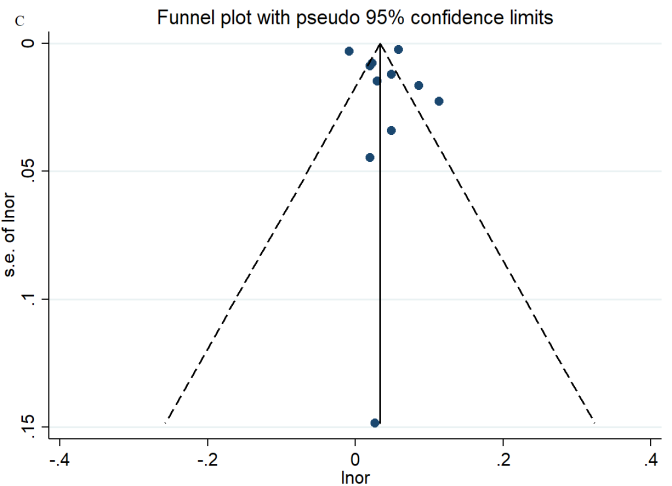

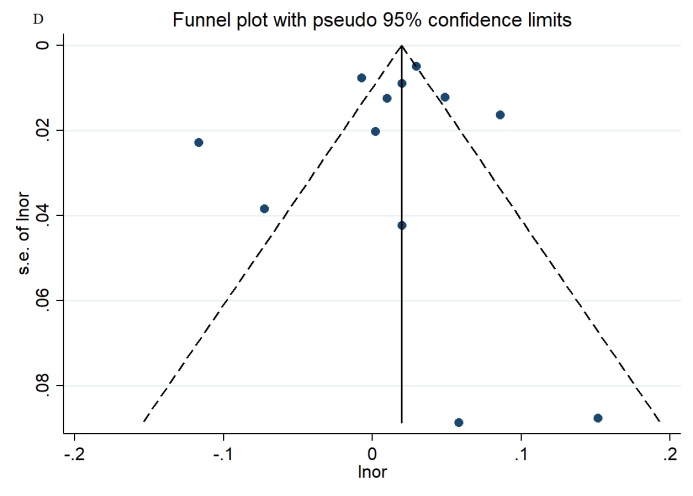


**Figure S5 Funnel plot of the association between ozone exposure and preterm birth in pregnancy. (A) Ozone exposure as a continuous variable in whole pregnancy; (B) Ozone exposure as a continuous variable in early pregnancy; (C) Ozone exposure as a continuous variable in middle pregnancy; (D) Ozone exposure as a continuous variable in late pregnancy.**


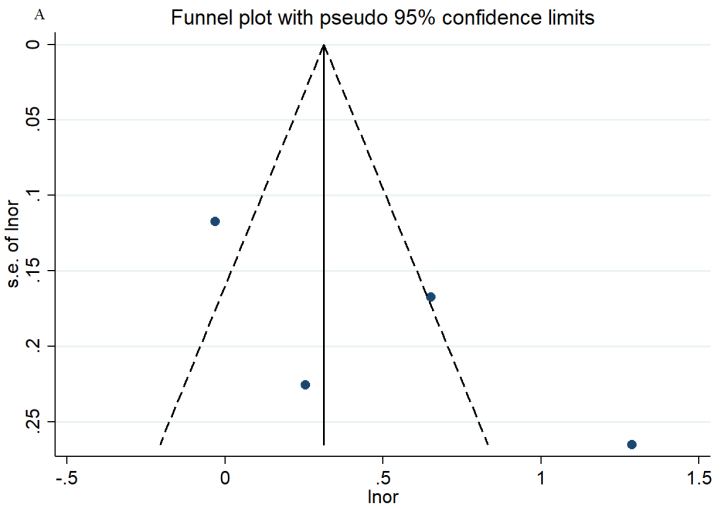

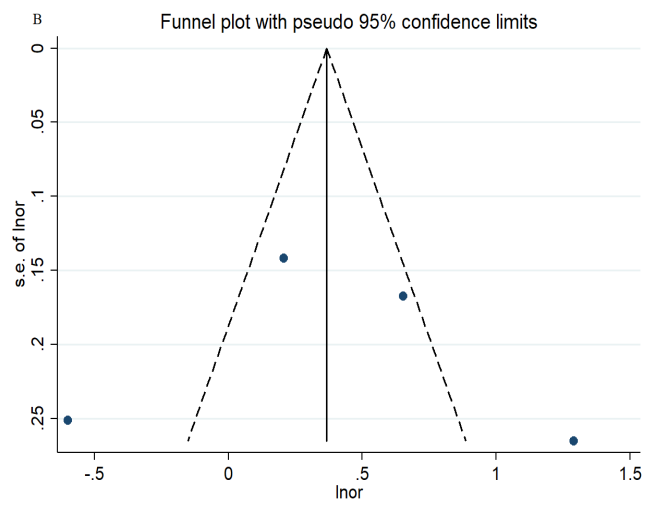


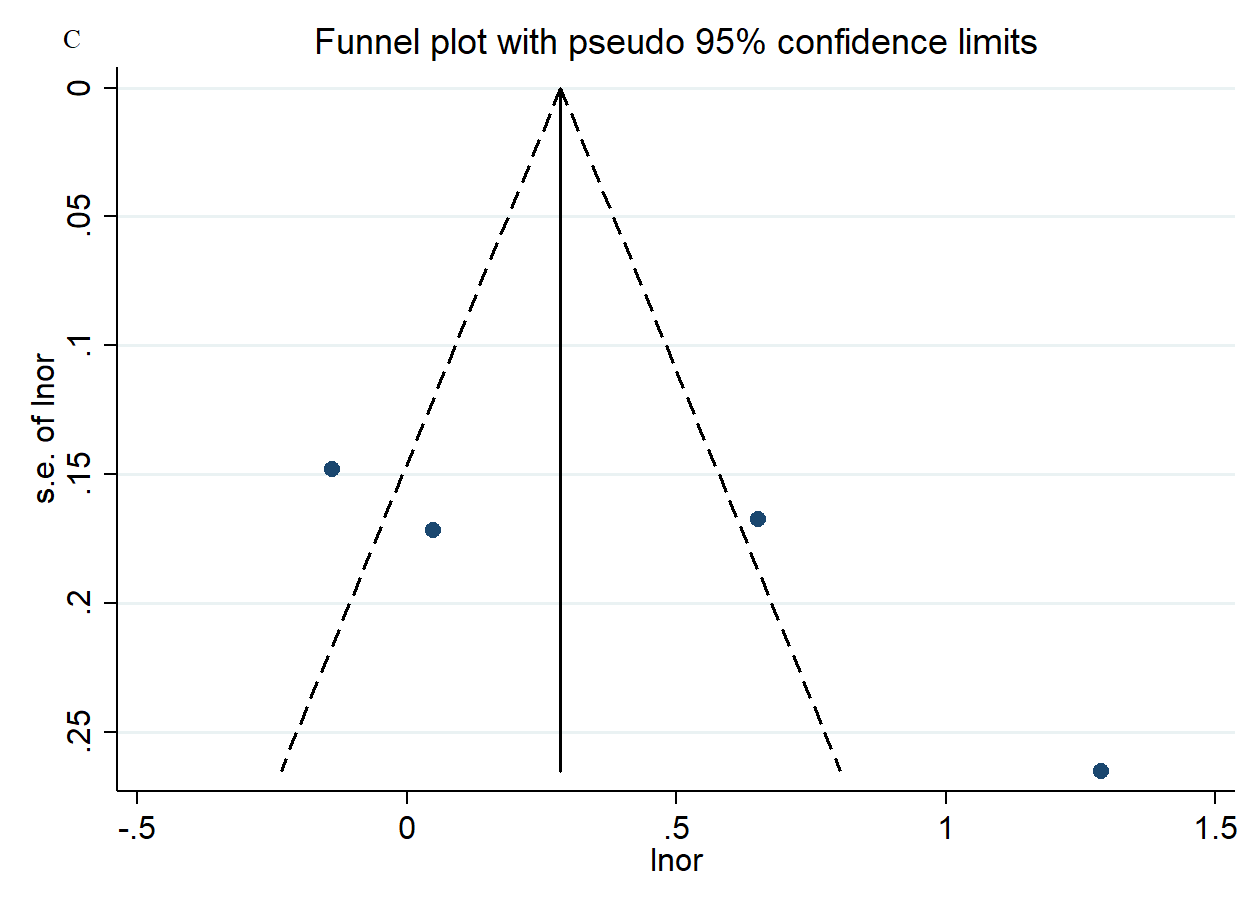

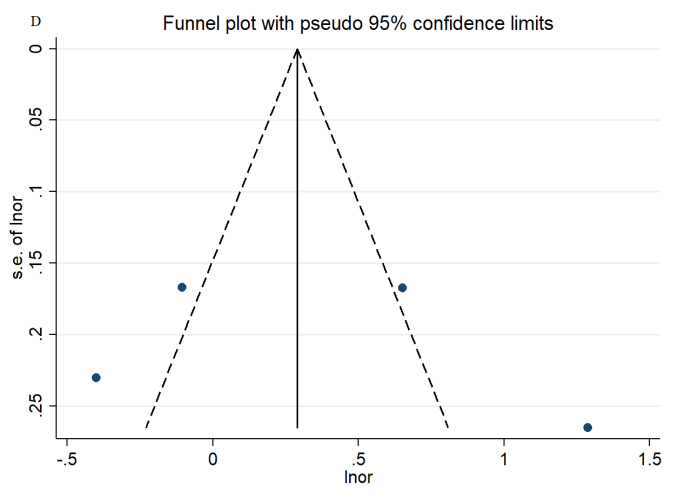


**Figure S6 Funnel plot of the association between ozone exposure and preterm birth in pregnancy. (A) Ozone exposure as a categorical variable in whole pregnancy; (B) Ozone exposure as a categorical variable in early pregnancy; (C) Ozone exposure as a categorical variable in middle pregnancy; (D) Ozone exposure as a categorical variable in late pregnancy.**
